# Life Story Work as an inclusive methodology to explore identity in intellectual disability

**DOI:** 10.3389/fpsyt.2026.1784316

**Published:** 2026-03-26

**Authors:** Araceli Arellano, Sarah Carrica-Ochoa, Olga Lizasoain

**Affiliations:** 1 School of Education and Psychology, University of Navarra, Pamplona, Spain; 2 National Distance Education University, Associate Center, Pamplona, Spain

**Keywords:** disability identity, identity construction, inclusive-oriented narrative approach, intellectual disability, life stories, Life Story Work, narrative research

## Abstract

This study explores how young people with intellectual disability (ID) construct and negotiate their identities through life stories, adopting a narrative perspective that foregrounds individuals’ own voices and lived experiences. Using a qualitative narrative research design grounded in Life Story Work (LSW), data were generated with eight individuals with ID through in-depth interviews, participant observation, photographs, and personal documents. An integrated analytic approach combining thematic and narrative analysis was applied to identify transversal patterns across narratives while preserving the internal coherence and temporal structure of each individual life story. Findings show that participants primarily defined themselves through roles, relationships, interests, values, and personal characteristics rather than through diagnostic labels, with disability emerging as a contextual dimension of identity rather than its defining core. At the same time, narratives revealed ongoing negotiations between self-perception and externally imposed meanings of disability, particularly in relation to social stigma and others’ attitudes. Family relationships and a strong sense of belonging played a central role in fostering positive identity construction, while life stories documented identity as a dynamic and evolving process shaped by key life transitions. Overall, the study highlights the value of life stories as spaces for identity construction and resistance to deficit-oriented disability discourses, underscoring the potential of inclusive, narrative methodologies for advancing more person-centered and socially just understandings of identity.

## 1 Introduction

People with intellectual disability [ID] do not constitute a homogeneous group; rather, each individual experiences and responds to life events in a unique way. Moreover, a person’s identity transcends their condition and cannot be reduced to the characteristic of disability. [The term intellectual disability is used following the DSM-5-TR (APA, 2022), where it is classified within the group of neurodevelopmental disorders, to ensure objectivity in the inclusion criteria. Beyond this, our conceptual stance aligns with the AAIDD’s multidimensional model, which underscores that functioning results from the interaction between individual characteristics, environmental conditions, and—most importantly—the provision of individualized supports that enable participation, autonomy, and identity development] (1) .

In recent years, the need to recognize and value the individuality and subjectivity of the experiences of people with ID—while also addressing their collective needs—has become increasingly evident (2). Within this recognition of individual life trajectories, people with ID themselves assume a central role. These accounts also resonate with the broader history of institutionalization and deinstitutionalization experienced by people with intellectual disabilities (3). Research processes are increasingly prioritizing their voices through inclusive and participatory approaches (4, 5). Within this framework, questions arise about how people with ID and other neurodevelopmental disorders (such as Autism Spectrum Disorder) construct their identity. From a theoretical perspective, identity is conceived as a dynamic process of self-discovery: who I am, with whom I relate, where I am situated, and what my story is. McLean (6) understands identity as a constantly evolving narrative that integrates new characters and plot turns, and can only be understood discursively. From Erikson’s (7) psychosocial theory, identity development extends throughout the entire lifespan, although it becomes particularly salient during adolescence. According to Erikson, identity results from a dynamic process in which the individual integrates their past, present, and future. Moshman (8) defines identity as an *explicit theory of oneself as a person* (p. 78), emphasizing its reflective nature. Identity, therefore, is flexible, changing, and constructed within specific social and cultural contexts. Historically, these contexts have placed disability in the realm of abnormality, deficiency, and personal tragedy (9, 10). For decades, dominant models, particularly the medical model, have defined disability as an individual defect to be corrected or compensated for, eclipsing other dimensions of identity and reinforcing stereotypes. Being labeled as a “person with a disability” can profoundly affect self-perception and sense of self due to the stigma and exclusion this population has historically experienced (11). From a narrative perspective, these dominant discourses not only shape social representations of disability but also delimit the identity resources available to individuals, influencing how they make sense of themselves and their life experiences (12, 13).

In contrast to hegemonic narratives centered on pity, heroic overcoming, or victimization (9), alternative proposals have emerged, such as the affirmative model (14) or the disability pride (15), which recognize disability as a legitimate way of being in the world and as a potential source of values and meaningful experiences. Acceptance of one’s own disability as an intrinsic trait has been associated with more positive relationships, a greater sense of purpose, and improved well-being and quality of life (16). Identity, therefore, is not constructed in isolation but in constant dialogue with the messages, attitudes, and relationships present in one’s environment. Language, diagnostic labels, and social expectations play a decisive role in how people with disabilities perceive themselves (17). Devaluing terms or stigmatizing representations directly affect the possibility of developing a positive identity. Studies on *disability identity* (18, 19) indicate that this process involves ongoing negotiation between personal self-perception and the social meanings attributed to disability. A sense of belonging, experiences of inclusion, and opportunities to tell one’s own story—using communication forms preferred by the individual—emerge as essential elements in strengthening a positive and integrated identity.

In this context, it becomes essential to facilitate opportunities for people with disabilities to tell their own stories—something historically overlooked, despite pioneering examples such as *The World of Nigel* Hunt (20), *Tongue Tied* (21), or *A Price to be Born* (22). In recent decades, the power of personal narratives has been increasingly used to improve support for people with disabilities. Under terms such as *life story work*, *life story*, or *life narrative*, this perspective recognizes the transformative value of personal storytelling in understanding identity and human experience (23). Narrative studies (24, 25) have shown that these stories enable people to construct meaning about themselves, express desires for recognition and self-determination, and challenge imposed categories. Listening to people with disabilities talk about their lives can be a powerful means of empowerment (25). Furthermore, the perspectives of people with disabilities about themselves often differ from professional or family interpretations. Understanding their narratives allows us not only to explore the multiple ways of living and understanding disability but also to critically examine the social structures that shape these identities.

In line with this perspective, identity as a process of meaning−making articulated through personal narratives, we conceptualize Life Story Work [LSW] not simply as a technique for eliciting information, but as a relational and interpretive practice that enables individuals to make sense of their experiences, relationships, and aspirations. This framing anticipates the methodological role of LSW in supporting participants to construct and communicate their autobiographical meanings.

Despite the potential of narrative approaches to deepen understanding of the experiences of people with disabilities, few studies have directly addressed identity construction through their own life stories. In response to this gap, the present study seeks to answer the following research questions: How do people with intellectual disability talk about their lives through LSW? What identity-related stories can be co−constructed from their narratives? What themes and narrative structures emerge from these co−constructed life stories? This research aims to explore how people with ID construct their identities through their individual experiences, recognizing the uniqueness of each story and the complexity of their identity processes. The research presented here also invites us, as researchers, to reflect on our role in constructing and representing knowledge about the lives of people with disabilities.

## 2 Method

### 2.1 Methodological approach

This study employed a narrative research methodology, an approach previously used to explore the identity of people with disabilities (26) and recognized as an ethical framework when working with this population (25). Within this approach, the researcher’s interest focuses on understanding the meanings individuals attribute to their experiences, as expressed through the ways they talk about the events of their lives. In this study, *narrative* is understood as any account of one’s life—spoken or written—produced by the person who is the protagonist of the story (27). Narratives are therefore conceived not merely as descriptions of events, but as meaning-making processes through which individuals actively construct, negotiate, and reinterpret their identities over time (27, 28).

In this study, narrative inquiry constitutes the overarching methodological and analytical framework, guiding the interpretation of how individuals construct meaning and identity through stories. Within this framework, Life Story Work (LSW) was used as a narrative facilitation tool that enables the co-production of autobiographical materials. While narrative inquiry provides the epistemological foundations and analytic procedures, LSW offers the practical structure through which participants generate the narratives that are later analysed.

Within this framework, the research employed LSW as a methodological tool for constructing participants’ narratives, following the contributions of Hussain and Raczka (29) Ledger et al. (30) Berger (31), and Hewitt (32). This tool is particularly relevant to achieving research guided by inclusive principles (5), as it promotes the expression and active participation of individuals in the generation of knowledge about their own lives. Furthermore, it is conceived as a method that respects and promotes the right to self-determination of people with ID enabling the recovery and recognition of their voices in understanding their personal histories (33). According to McKeown et al. (34), LSW is defined as:

A form of intervention (…) encompassing a range of terms/interventions, for example biography, life history, life stories (…) and usually results in a product, for example a story book, collage, notice board, life history/biography, or tape recording.

It is important to clarify that in this study LSW was not implemented as a therapeutic or clinical intervention, but as a research-oriented narrative facilitation strategy. While LSW has frequently been used in care or clinical contexts, in this project it functioned as a structured framework to support autobiographical narration and meaning-making for research purposes (30, 35).

Within this research-oriented framework, LSW was not used solely as a data collection technique, but as an interpretive and relational process that supported participants in organizing their experiences into coherent narratives. This process involves working collaboratively with the person to explore various aspects of their life—both general (experiences, relationships, and emotions) and more specific (places lived, personal data, significant dates, or daily activities)—thus contributing to a comprehensive and situated understanding of their biography (36).

The project began in 2021, with the creation of an interdisciplinary team involving university researchers and professionals from one mainstream school, two special education schools, and two associations providing services for adults with ID. From the outset, these professionals participated as co-researchers, actively contributing to the design, planning, and development of the study. Throughout the project, regular meetings were held and qualitative research memos were used, to document the process and methodological decisions. In addition, a series of common guidelines were established to guide the implementation of LSW and to ensure the ethical coherence and participatory consistency of the study (see **Table 1**). Data collection took place during 2022 and 2023, and in 2024, the data analysis and construction of the resulting life stories were carried out. The process culminated in the construction of an individual life story for each participant. These stories were not mere chronological compilations of facts, but rather jointly elaborated narratives created by the participant and the research team. Multiple data sources—oral accounts, visual materials, photographs, diaries, and field observations—were integrated to create narratives that express each person’s identity, relationships, and life trajectories.

**Table 1:** Methodological guidelines for the Life Story Work process.

Dimension	Description
Objective	To construct the life story of people with intellectual disabilities through a process of collaborative and meaningful interaction.
Context	Data collection is carried out mainly in the participant’s natural environment fostering a familiar and safe atmosphere.
Nature of information	Both objective aspects (events, dates, data) and subjective aspects (values, feelings, perceptions, and personal interpretations) are addressed.
Privacy and consent	The life story may acquire a public dimension, but the participant always decides freely which aspects to share and which to keep private.
Flexibility and support	Materials and activities are flexible and adapted to each participant’s abilities, communication skills, memory, introspection, and life stage.
Complementary informants	While external informants may be consulted, priority is always given to the meaning the person themselves attributes to their experience.
Role of the researcher	The researcher acts as a facilitator, accompanying and supporting the narrative process without judging the participant’s experiences or interpretations.

### 2.2 Recruitment

An invitation to participate in the study was extended to a group of professionals from several organizations. Once the professionals responsible for the services became familiar with the project, they were the ones who contacted the potential participants and their families to inform them about the research. In some cases, an introductory session about the project was held with the direct participation of the researchers. Information sheets and consent forms were provided to all potential participants and their families, and these were also explained verbally to ensure full understanding. The inclusion criteria were as follows: (a) adolescents and adults with ID who had oral language skills; (b) all participants had to be known by the professionals for at least six months; (c) at least one family member of each participant had to be available to take part in at least one interview with the researchers; (d) participants needed to be available to engage in the research process for a minimum period of nine months. A collaboration agreement was established with five centers. Each of them contributed two participants, except for one mainstream school, from which 9 students took part. In total, 17 people voluntarily agreed to participate in the study after being fully informed about its aims and procedures. From the total number of participants who initially agreed to take part in the study, only those who completed the entire LSW process—including the reading and validation of their final life story—are included in the data presented in this paper.

Written informed consent was obtained from their parents, as well as from the corresponding educational institutions. In addition, assent was sought directly from each young participant. The research project was explained to them in accessible language by both teachers and members of the research team prior to the interviews. Participation was voluntary at all times, and researchers remained attentive to verbal and non-verbal indications of willingness to continue throughout the process.

To ensure confidentiality and privacy, all names used to identify participants in this article are pseudonyms, selected in accordance with the confidentiality agreement signed within the framework of the study. The pseudonyms were chosen by the researchers to be common, maintaining the gender and age diversity represented in the actual sample. The pseudonyms bear no relation to participants’ real names or identifying characteristics, thereby safeguarding their anonymity and privacy throughout the research process.

Of the 17 individuals who initially agreed to participate, eight completed the full LSW process, including validation of the final narrative text. Reasons for non-completion were related to availability and time constraints rather than distress associated with participation. This attrition is acknowledged as a limitation.

### 2.3 Participants

Participants presented diverse neurodevelopmental profiles; while all had an ID diagnosis or were described by families and professionals as having significant intellectual and adaptive support needs, some also had additional diagnoses (e.g., ASD) or rare syndromes.

The participants were four women and four men between the age of 13 and 32, who had neurodevelopmental disorders, all of them had a diagnosis of ID. The participants’ characteristics at the beginning of the study are presented in **Table 2**. In all cases, family members were also involved (eight mothers, two fathers), and four key professionals (special education teacher, school psychologist, psychologist and occupational therapist).

**Table 2:** Participants’ characteristics.

Pseudonym	Age	Gender	Living situation	Educational/support context	Diagnosis
Emma	23	Woman	With parents and brother	Day center for adults with disabilities	ID
Mael	25	Man	With parents	Occupational center	Down syndrome
Sofia	16	Woman	With parents and brother	Mainstream school (self-contained classroom)	ID
Elias	14	Boy	With parents and siblings	Mainstream school (self-contained classroom)	PDD (family report); ASD/ID suspected
Maya	14	Girl	With parents and sister	Mainstream school (self-contained classroom)	8p23 deletion syndrome
Max	16	Boy	With parents	Mainstream school (self-contained classroom)	Williams syndrome
Leo	13	Boy	With parents and sister	Mainstream school (self-contained classroom)	ASD and ID
Eva	32	Woman	Supported living apartment	Day center for adults with ID	Autism, ID, oppositional defiant disorder

Family members contributed complementary accounts that helped contextualize life events and support chronological structuring of the narratives. However, the individual remained the central narrator. The final life story texts were reviewed and validated with them to ensure that their perspective was preserved and that family recognize their relatives. Given the biographical and longitudinal nature of life story construction, family members provided temporal and contextual scaffolding, particularly for early childhood events or experiences not directly recalled by participants. However, the participant remained the central narrator, and the final life stories were always reviewed and validated directly with each participant.

### 2.4 Instruments

A qualitative multi-method approach was adopted, combining individual work (one-to-one) with small-group sessions in which several participants worked together. Depending on each participant’s profile, several qualitative techniques were employed, including:

- In-depth interviews: conducted with participants and their family members. Data were collected using an interview guide, an audio recorder, and researcher field notes.
- Participant observation: was carried out during small-group sessions in which participants worked on the development of their personal timelines, mapped significant people and places, and shared personal objects and photographs. Data were documented through a field diary, an observation protocol, and photographic records maintained by the researchers. Observational notes were kept to document the process and provide contextual background. They were not analysed systematically but supported the interpretation and contextualization of participants’ narratives.
- Review of personal objects and documents (photographs, drawings, diaries, certificates) was used to explore the personal meanings that participants attributed to the items they selected. Photographs and personal documents were treated as narrative artefacts and analysed in relation to the meanings attributed to them by participants during the storytelling process, rather than as standalone visual data.

This combination of techniques and instruments allowed for a rich and multi-source understanding of each participant’s life story, ensuring that both subjective experiences and contextual information (educational settings, place of birth, family composition, among others) were captured. Given the complexity of the process, stemming from the combination of diverse techniques, supports and participant profiles, a detailed guide was developed for all researchers, specifying the techniques, instruments, and materials to be used with each participant. This guide ensured methodological consistency while allowing flexibility and adaptation to individual needs. For example, we used tools drawn from Person−Centered Planning (PCP), such as different versions of Making Action Plans (MAPS) and Circles of Support (37), to suit participants with diverse profiles, and specific materials were prepared for some group sessions—such as visual aids for constructing a personal timeline with photographs in a mural format. These adaptations supported meaningful participation and helped capture the unique ways in which each person expressed their experiences and memories. Trusted professionals assisted only in ensuring understanding, adapting materials when required, and facilitating communication. They did not intervene in shaping the narrative content.

### 2.5 Data analysis

This study adopts a narrative design, as individual life stories were constructed and validated as coherent narrative wholes. To identify shared identity dimensions across cases, a cross-case thematic analysis was subsequently conducted. This integrated narrative–thematic strategy allowed us to preserve the integrity of each life story while also identifying transversal patterns across participants.

The analysis followed an integrated approach combining thematic analysis (38) and narrative analysis (39). This integration was sequential and iterative: thematic analysis was first used to identify transversal patterns across narratives, which were subsequently interpreted through narrative analysis to preserve the internal coherence and temporal structure of each individual life story, as well as the processes through which identities were constructed over time.

The analytic process unfolded in two interconnected phases. First, all materials—including interview transcripts, field notes, photographs, and personal documents—were repeatedly examined as narrative texts to identify initial units of meaning related to identity and life experiences. These units were inductively coded following the principles of thematic analysis, resulting in a preliminary set of descriptive codes. In the second phase, these codes were reinterpreted within the broader structure of each participant’s life story. Narrative analysis was used to examine how events were sequenced, how participants positioned themselves within their narratives, and how turning points, tensions, and resolutions contributed to identity construction. This dual approach enabled the integration of transversal depth (shared themes across participants) and longitudinal depth (the evolution of identity within individual narratives).

The coding process was iterative and collaborative. Data analysis was conducted as an iterative and reflexive process. Coding and category development evolved through successive cycles of independent analysis, comparison, discussion, and refinement among the research team. Two researchers independently coded two complete life stories to generate an initial pool of codes. By “complete life stories,” we refer to the consolidated case narratives produced at the conclusion of the Life Story Work process. These narratives integrated interview transcripts, visual artefacts, field notes, and contextual contributions from significant others, and were validated with each participant before formal analysis. These were then compared, merged, and refined through consensus discussions, resulting in a preliminary coding framework. The first and second authors conducted the primary coding process. After independently analyzing the pilot cases, they met to compare interpretations, discuss discrepancies, and collaboratively refine the preliminary analytical framework. The third author participated in subsequent analytic discussions, contributing to the critical review of emerging categories and supporting reflexive validation. This framework was subsequently applied to the full dataset, allowing additional subthemes to emerge. Previously analysed cases were revisited after each modification to ensure conceptual coherence and consistency across narratives. Subthemes were defined when (a) they recurred across at least three narratives, or (b) they represented conceptually significant dimensions of identity construction. A final axial coding ensured internal coherence within each theme and clear conceptual boundaries between them. To enhance credibility, triangulation was conducted at three levels: researcher triangulation, data triangulation, and participant validation (member checking).

While initial coding was inductive and grounded in participants’ narratives, interpretation of the final themes was informed by relevant literature on disability and identity development. The analytic approach can therefore be described as primarily inductive, complemented by theoretically informed interpretation. For example, initial descriptive codes capturing experiences such as feeling loved, being misunderstood, or perceiving the family as a refuge were later grouped under the broader thematic category “Narratives of Belonging or Exclusion”. Similarly, codes related to the diagnostic experience (e.g., the diagnostic moment, medical reports, or the act of naming the condition) were integrated into the theme “Diagnosis and Labels”. In line with qualitative research standards, rigor was addressed through credibility, dependability, confirmability, and transferability (40), rather than statistical validity or generalizability.

### 2.6 Ethical considerations

Ethical approval was obtained from the Ethics Committee of the University of Navarra. The research team ensured that the life story process remained clearly differentiated from therapeutic or clinical interventions. Participants and families were informed that the purpose of the project was to generate knowledge through narrative research. No psychological treatment or intervention was provided as part of the study. Particular attention was paid to ensuring that all participants fully understood the purpose and procedures of the study prior to providing informed consent. A respectful and collaborative research approach was adopted, fostering an environment in which participants felt comfortable sharing their life stories. Participants were reminded that they could stop, take breaks, or skip any topic at any time to ensure emotional safety. The researchers’ experience working with individuals with intellectual and developmental disabilities further contributed to the safe handling of sensitive memories. No trauma−triggering reactions occurred during the study.

Researchers prioritized participants’ preferred modes of communication and allowed sufficient time and support to facilitate expression. Anonymity was ensured throughout the research process by removing potentially identifying information and using pseudonyms in all outputs. Additionally, data-separation procedures were implemented: interview transcripts and field notes were stored independently for each participant, and no family member or professional had access to any personal interview content unless the participant explicitly chose to share it. This ethical framework sought to uphold participants’ dignity, self-determination, and well-being, in line with the principles of inclusive and participatory research.

## 3 Results

Six overarching themes were identified across participants’ life stories: (1) Self-definitions and External Views; (2) Identity Transformations; (3) Relationship with the Environment; (4) Narratives of Belonging or Exclusion; (5) Diagnosis and Labels; and (6) Roles and Personal Identities Beyond Disability. Each theme captures a central dimension of identity construction emerging from the cross-case analysis.

The six final themes were selected because they captured core dimensions of identity construction identified in the literature on disability identity and narrative research (9, 17, 18) and emerged consistently across the empirical material. These narratives are not verbatim transcripts but constructed texts that integrate the participant’s voice with the researchers’ interpretative lens. This section includes three types of excerpts: (a) direct quotations from participants (either written or taken from the interviews), (b) statements contributed by family members or other significant persons, and (c) fragments from the resulting life story texts. These different voices are identified in the text to ensure clarity for the reader.

### 3.1 Self-definitions and external views

This theme captures how individuals describe themselves and how they are described by others. It includes expressions of personal identity, traits, and values, as well as references to expectations imposed by family, professionals, or society.

In the analyzed stories, personal descriptions emerge that reveal how participants perceive themselves at different moments in life. These descriptions take diverse forms; they are expressed through words and images or photographs. On the other hand, representations of the person with a disability by their family members and supporting professionals are also documented. The terms used by individuals with disabilities to describe themselves include:

*friendly, hardworking, if someone has difficulty, I help them* (Sofía); *slim, pretty, hardworking*, sp*orty, happy, cheerful, kind, fun, sensitive, impatient, nervous, talkative* (Maya); *hardworking, winner, happy, excited, I give a lot of love* (Emma); *cheerful, I like to help, hardworking, a machine* (Mael).

Family members tend to highlight traits such as *cheerful, stubborn, empathetic, playful, happy, fun-loving* (Sofía); *survivor, vulnerable, fun, inquisitive, affectionate, hardworking, dancer, sufferer, nervous, impatient, sociable* (Maya); *bossy, likes to be the center of attention, imaginative, lazy* (Emma); *sensitive, in need of affection, empathetic* (Elias); *stubborn, impulsive, radiant* (Eva); *vulnerable, affectionate* (Mael); *pure love, annoying, affectionate, social, musical, flattering, noble, authentic, hardworking, captivating* (Max).

Notably, none of these groups spontaneously use diagnostic or disability-related terms as the primary descriptors of the protagonists. However, such labels do appear at different points in the narratives, often integrated as part of their broader identity. In response to the question “Who am I?” the protagonists’ descriptions do not prioritize this aspect, as reflected, for example, in the following excerpt written by one of the participants:

*I am 1.59 m tall. I weigh 53 kg. My skin color is light. My eye color is brown. My hair color is dark brown. I don’t wear glasses or braces, for now. My shoe size is 41, my pants size is 14–16. My personality: I am very introverted. I like to be calm and alone, but I also like going to the movies with my dad. I have many friends at school. I have a cat and a little dog.* (Max)

When asked *“*How do you think others see you?*”* responses generally reflect positive and affectionate views from close relationships, as illustrated by Sofía’s words:

*My parents say I am studious and hardworking.*

*Mark likes me and says I am a good friend.*

*Fran says I’m really nice.*

*Mom says I’m her sweetheart, good, cheerful, funny, and that I care about others.*

*Dad thinks I’m cheerful, stubborn, responsible, tech-savvy, with common sense, religious, and a night owl.*

*My brother says I’m smart, good, a great sister, kind, that I always have a smile, always there. You’re very intelligent. You make me mad sometimes. Funny and nice. Make people laugh. Smiley. Pretty.*

*My other brother says: clever, with a witty sense of humor and cuddly. He gives me lots of hugs.*

*Grandma says: responsible, studious, hardworking, and very loving to her grandma.*

However, some narratives also acknowledge negative societal perceptions, as Mael notes:

*People know, even if they don’t really know us, who we are. They know us through Facebook and social media. They look at us and sometimes visit. It’s the same as some who have cerebral palsy, others don’t. They think we don’t think, and sometimes they ignore us, but we do think—just differently. All people with disabilities are different, and each one can do some things but not others*. (Mael)

### 3.2 Identity transformations

This theme refers to changes or shifts in how individuals perceive and narrate their identity over time. It includes turning points, life transitions, and reinterpretations of past experiences that lead to a redefinition of the self. Participants narrate life transitions—such as changes in educational settings, moments of diagnosis, or significant losses—that mark turning points in their self-perception. These transformations reflect adaptability and show how identity is reconstructed over time. They also highlight the dynamic nature of identity.

Within this code, three subthemes are identified. The first one, stages and transitions, refers to the passage between life stages (childhood, adolescence, and youth). This is the case of Eva’s life story, whose narrative is structured into three stages that reflect three different ‘Evas.’ As her mother says: *there is not just one daughter, there are many more.* As mother´s Eva shares, there are three distinct daughters, as if each stage of life, upon ending, had given rise to a new birth. Moreover, Eva’s narrative includes three poems written by her mother, one for each stage of her life (childhood, adolescence, and her current adult life). Transitions are mostly described by family members, although participants understand the concept of change, sometimes needing to explain it through observable traits (such as physical changes). The importance of making these processes visible is evident, through work with photographs or memories made explicit with objects.

The second subtheme, turning points, encompasses events that mark significant shifts in the biographies, among which moments related to the time of diagnosis and changes of educational setting stand out. Related to the medical field, Max’s experience stands out. At the age of 13, he received a medical report containing the words that would change the course of his story. This is illustrated in the following excerpt from her narrative, which cites the results of that report:

*Summary of tests: The hybridization pattern is compatible with the presence of a deletion in the critical region of Williams syndrome.*

*Clinical judgment: Williams-Beuren syndrome.*

After the multiple comings and goings described so far, a suspicion arose that led doctors to conduct an in-depth genetic study. And there it was: a deletion on chromosome 7. In short, part of Max’s genetic material was ‘lost,’ specifically on chromosome 7—the missing piece of the puzzle. Along with the medical report, Max keeps a photograph of that moment. In it, he is surrounded by the specialists who carried out his assessment and diagnosis. Everyone is smiling, and Max is making the victory sign with his left hand. *Finally! We have a name and surname for everything that is happening to me.* And that is how Williams syndrome became part of his identity. On the very day of the diagnosis, his parents learned that the International Update Conference on Williams-Beuren Syndrome, organized by the Spanish Williams Syndrome Association, would soon take place. They recall it as follows:

*It was spectacular. Newly diagnosed, having that information … Suddenly, a person with Williams syndrome, 20 years old, comes up and talks to you. We cried. It was seeing reality in full force—it gave us such a boost.* (Max’s mother)

Finally, the subtheme growth refers to processes of personal development through which new narratives about oneself are constructed, generally in a positive sense. In this sense, the narratives reflect everyday transitions related to small achievements (for example, gains in autonomy or maturity) that allow them to grow not only at an individual level but also as a family: *we have moved from loneliness and bitterness to seeing the sun* (Leo’s mother).

### 3.3 Relationship with the environment

This theme captures how individuals relate to their social, physical, and institutional environments. It includes interactions with contexts and how these relationships influence identity construction. Within this code, two fundamental subthemes emerge: family and the school environment. The narratives reveal how relationships with family and community spaces shape the sense of belonging and self-esteem. Experiences of support, recognition, or exclusion in these contexts appear as key elements in the consolidation of a positive identity or, conversely, in the experience of vulnerability. In most of the stories, the family appears as the main source of emotional support. Sofía begins her narrative by describing her home: *In this family live five people: mother L., father L., brothers A. and M., and sister Sofía.* This way of introducing herself shows that identity is constructed relationally, not individually: the self is expressed within the ‘we.’ Maya proudly says: *I have a very beautiful family.* Families describe processes of mutual adaptation: *The family has adapted to him*, says Leo’s mother, highlighting that inclusion begins at home. In some cases, the family becomes a refuge from external misunderstanding; in others, a platform for autonomy.

The school environment occupies a central place. The narratives of families and young people reveal trajectories marked by the search for spaces that recognize their abilities and needs. School is described as an environment that is sometimes welcoming, sometimes hostile. Maya’s mother expresses relief that her daughter has found her place: *It’s her* sp*ace*, referring to the self-contained classroom^1^. In contrast, Elía´s father recalls with difficulty: *The hard part comes when crossing the door of the classroom.* When professionals understand and provide support, families speak of ‘flourishing’: *At school, when they understand him, he flourishes* (Leo´s mother). Conversely, when institutional responses are rigid or deficit-oriented, young people experience withdrawal or frustration.

Family and the educational system act as key pillars, but they are not the only ones. Identity is also shaped in other spaces. In many narratives, school experiences are complemented by participation in leisure and friendship contexts, which serve as alternative places of belonging. Sports clubs, leisure associations, or art workshops become environments where young people can interact on more equal terms, free from labels or academic judgment. In these spaces, friendships arise from shared interests rather than differences, reinforcing self-confidence and a sense of personal worth. Several participants mention friends from associations or activities as the most significant relationships in their daily lives. Social interaction in these contexts has a restorative effect: they are spaces where they can simply be themselves, beyond what they represent. However, in most cases these leisure spaces are structured and framed within the associative movement of people with ID; experiences of inclusive leisure are scarce.

### 3.4 Narratives of belonging or exclusion

This theme identifies experiences of inclusion, exclusion, acceptance, or rejection. It reflects how individuals experience their place in social groups, communities, or society at large. Belonging is not reducible to simply “being in” a group; rather, it entails being recognized, cared for, and listened to. Conversely, exclusion is described as indifference or a lack of understanding. The narratives show that belonging is relationally constructed in families, schools, and community spaces, and that the same settings can alternately foster inclusion and reproduce forms of exclusion.

Participants frequently describe exclusion as moments of pain and vulnerability tied to stigma and misrecognition. As Elias writes: *What I like least is feeling alone, being looked at with contempt*. Similarly, Leo’s mother highlights the social roots of misunderstanding: *People do not always understand his reactions*. These accounts underscore that exclusion often stems from limited empathy and interpretive charity, which can generate isolation even when formal support is in place. In this sense, exclusion is experienced as a relational failure rather than merely a lack of access.

In contrast, participants also report powerful experiences of inclusion anchored in affection, recognition, and active participation. Elias describes how his sense of belonging strengthens in community spaces: *When I am with the giants, I feel very happy* (referring to a local cultural troupe). Such contexts enable participants to be seen not through the lens of difference but through shared practice, joy, and contribution. Families likewise frame inclusion as sustained emotional connection rather than resources alone. As Maya’s mother notes: *She needs to feel very, very, very loved*. Max’s mother adds: *He wants everyone to be his family*, while Eva’s mother emphasizes explicit affection: *We have loved her deeply and reminded her countless times how beautiful she is—so much so that she now sees herself that way.* Mael emphasizes: *They take care of me, give hugs and lots of affection—it’s true love* and *My relatives are the most important; I love* sp*ending time with them*. Across accounts, emotional bonds and recognition emerge as the deepest expressions of inclusion. Narratives consistently position emotional validation—feeling loved, valued, and accompanied—as a necessary condition for building a positive identity. Validation does not simply buffer adversity; it actively enables participation, voice, and a sense of being entitled to belong. Where validation is absent, participants describe internalizing stigma and withdrawing; where it is present, they depict greater confidence in navigating family, school, and community settings.

Specialized structures—such as self-contained classrooms, associations or adapted leisure groups—are described ambivalently. On the one hand, they provide safety, friendship, and understanding; on the other, they can reinforce a sense of difference. As one mother remarks about her daughter’s center: *It’s her place*, capturing the relief of finding recognition. At the same time, another parent cautions: *Sometimes I think they live too much among equals*, articulating the tension between protection and isolation that many families navigate. These accounts highlight how belonging within “special” spaces can coincide with partial exclusion from “ordinary” environments.

### 3.5 Diagnosis and labels

This theme refers to how diagnostic terms, medical labels, or societal classifications are mentioned, interpreted, accepted, resisted, or redefined in the life story.

Diagnostic terms and clinical labels generate meanings that affect identity (9). The life stories of some participants reflect how the diagnostic processes unfolded and to what extent classification within a particular system of categories has impacted multiple areas: family life, self-perception, sense of belonging, among others.

Different narratives were found in the experiences of individuals and their families. Family members—especially parents—describe these processes with greater intensity, associating them with negative feelings such as uncertainty or anxiety, and positive ones such as relief. One parent recalls:

*After endless evaluations, reports, and unanswered questions, the diagnosis arrived: autism* (Leo’s family, after a long trajectory of consultations.)

In the case of the protagonists of the stories, the diagnostic process does not appear explicitly in their narratives, although most of them do identify the labels that explain their condition. This dual reality became evident during the development of the life stories: on one hand, what parents recount about the diagnosis and how they have addressed it with their children; and on the other, how the protagonists themselves integrate that information into their personal narratives. For example, Mael oscillates between awareness and resistance:

*Sometimes he says he is special and knows he has Down syndrome; yet when he looks in the mirror, he insists he has no such features.* (Mael´s mother)

Maya integrates genetic information into her own narrative:

*My cells are different because they are missing a little piece … I’m clumsy in movements and in thinking* (Maya).

In Max’s life story, the diagnostic process acquires particular relevance, as reflected in the narrative excerpt developed from interviews and supporting documents. Excerpt from Max’s life story (final text):

For his parents, Williams syndrome is perceived as a natural part of Max’s life and personality, and this is how they convey it to him. Max knows and is aware that he has Williams syndrome and often refers to it in a positive way. However, in moments of anger or frustration, phrases appear that recall the stigma still surrounding the lives of people like Max *(I can’t do it, it’s because I’m stupid*)*!*. When we ask Max about Williams syndrome, he explains:

*It’s a syndrome that is like this [makes a W gesture with his fingers]; they are friends I have in an association where my parents could also be with other parents. They love me. They come from all over Spain—Málaga, Seville, Jaén. And what happened to me was hyperacusis; when I was little, noises bothered me, children bothered me, that’s why I wear earplugs. I had that, I still have it, but well…”* (Max).

These examples illustrate how diagnostic categories, while medically defined, become embedded in personal and family meaning-making. In many cases, diagnostic labels are assumed as part of identity—not from a clinical perspective, but as a way of understanding oneself in relation to the environment. This dialogue between family voices and individual voices reveals processes of shared meaning-making, where emotional support and the way information is communicated play a key role in shaping a positive identity.

### 3.6 Roles and personal identities beyond disability

This theme captures the various roles and identities individuals assume beyond the label of disability. It includes references to any other role that contributes to their sense of self and social participation.

In the analyzed stories, participants are described beyond their belonging to the population group with disabilities. The meanings they attribute to various roles—such as student, friend, sibling, youth—are explored. Including these types of roles in the narratives challenges stereotypes and helps promote a pluralistic view of people with disabilities.

The life stories reveal a rich diversity of roles and interests that shape identity beyond clinical labels. For instance, Elias’s narrative highlights his passion for communication and media:

*When he was little, I made him a microphone that said Radio Television … He broadcast everything, even the San Fermín festivities and football matches. It was spectacular.* (Elia´s father)

Later, Elias himself states: *I like journalism, I really like TV … For example, being a presenter of television programs. But for that I have to go to university and study that career.* Other roles emerge through hobbies and aspirations. Emma expresses her artistic and athletic interests: *I would like to take painting and art classes. I’m good at* sp*orts: ping pong, tennis, paddle, football, weightlifting, swimming, dancing.* Leo’s story illustrates multiple identities beyond disability, portraying him as a curious learner, a family member, and a sports enthusiast.

Excerpt from Leo’s life story:

Leo is the big-eyed boy who plays ‘pollito de la suerte’ while waiting in the doctor’s office. Leo is the older brother who teases little sister. Leo gathers chestnuts in the park and throws pebbles into the pond. Leo enters school happy and leaves happy. Leo is the guest of honor who walks through the university library and asks about history books. Leo is the patient who, from a very young age, undergoes tests, participates in evaluation processes, and answers questions over and over again about what he knows, perceives, feels, and learns. Leo is the observer of the bread slicer at the supermarket. Leo can recite the main countries and capitals of the world but needs hours of practice to brush his teeth properly or pack his school bag. Leo is a specialist in straight lines. In geography. In dictators. In NATO. In video games. Leo is a teenager who wants to study, work, and live with a girlfriend—preferably in a big city. Leo is the boy who plays sports twice a week with friends. Leo is the grandson who finds peace at his grandparents’ house and enjoys his space. Leo is the child who asks for a printed Wikipedia for Christmas. Leo is the super expert who looks for strategies to beat every video game that comes his way. Leo is the boy who talks to the spider on his terrace.

Mael projects his future through vocational and leisure roles: *My greatest wish is to be a carpenter*. His mother also identifies strongly with sports and faith: *He practices* sp*orts five days a week: swimming, athletics, football … He was runner-up in Spain in shot put. Religion is very important in his life; he loves the story of Jesus and Holy Week processions.*

Max’s identity is enriched by music and social participation: *He likes music, especially brass bands and flamenco … He collects festival programs, loves giants, and enjoys being the center of attention* (Max´s mother).

These examples illustrate how participants construct identities through roles that transcend disability, emphasizing hobbies, aspirations, relationships, and values. Such narratives challenge reductive views and underscore the importance of recognizing people with disabilities as individuals with multifaceted lives.

## 4 Discussion

This study explored how individuals with ID construct their identities through life stories, emphasizing identity as a dynamic, socially situated, and narratively mediated process (6–8). In relation to the original research questions, all the protagonists of the life stories talked about their lives and demonstrated the ability to recall and reconstruct aspects that were personally meaningful. Through these memories, they shape their identities and express who they are and how they feel about themselves. Each life story is unique, and the data reinforce the notion that identity is flexible and context-dependent (26). This diversity makes it difficult to fit life stories into categories. Both participants and their significant others referred to multiple, interwoven factors that contribute to identity—such as values, cultural background, social contexts—highlighting the complexity and overlapping nature of these dimensions.

However, some common themes emerge in the narratives and are consistent with findings from previous research on identity construction among people with intellectual disabilities (41, 42). First, the narratives reveal that identity extends well beyond the condition of disability, encompassing roles, interests, values, relationships, and personal aspirations. This finding challenges dominant approaches in disability identity research that place disability at the center of self-definition (10, 11). Similar to the study by Dorozenko et al. (43), the category of disability is not central to describing their identity. Instead, participants’ accounts suggest that disability often operates as a contextual dimension of identity rather than its defining core. This nuance enriches the existing literature by showing how individuals with ID actively resist or downplay disability labels in shaping their self-understandings, supporting previous evidence that disability is not always the most prominent identity marker (44).

Families, however, often assign a central relevance to their children’s condition, even while acknowledging other personal traits. Family narratives do not always coincide with the self-narratives of individuals, as recent research has shown (45). This divergence matters because parents frequently play a key role in reconstructing their children’s life stories, which can influence identity formation (46). Therefore, it is essential to ensure that these narratives remain as close as possible to the protagonists’ own words, respecting their agency and perspective (25).

At the same time, the findings highlight the persistent influence of social stigma and external perceptions. As Goffman (47) conceptualized, stigma operates as a social process that discredits individuals based on attributes deemed undesirable and shaping interactions. Participants’ narratives reveal an ongoing negotiation between self-perception and socially attributed meanings of disability, particularly in relation to experiences of being ignored, underestimated, or devalued (18). Notably, discomfort and distress were more often linked to others’ attitudes than to disability itself, reinforcing critiques of individual-deficit models and pointing instead to relational and contextual sources of vulnerability.

The findings reinforce the idea that identity emerges through ongoing dialogue with social environments and significant others, rather than being fixed or defined by diagnostic categories. In this sense, identity is shaped not only by personal experiences but also by the social meanings attached to disability, particularly how disability is understood and interpreted by close others (17). Taken together, these findings respond directly to our guiding research question by showing that identity construction among persons with intellectual disabilities emerges as a relational, evolving, and narratively mediated process rather than as a fixed outcome of diagnosis.

From a mental health perspective, these findings underscore the importance of moving beyond exclusively diagnostic-centered frameworks when seeking to understand the lives of people with ID. Narrative approaches such as LSW can complement clinical assessment by capturing subjective meanings, relational contexts, and sources of agency that are often overlooked in diagnostic practices. Incorporating these perspectives into mental health and psychosocial support may contribute to more person-centered, respectful, and recovery-oriented forms of care.

A strong sense of belonging emerged as a central protective factor in identity construction. Participants consistently described family relationships as spaces of emotional safety, validation, and recognition, where feeling loved, valued, and accompanied played a decisive role in fostering positive self-concepts. This finding aligns with the affirmative model of disability (14), which emphasizes the potential for meaningful and positive experiences within disability contexts, and with previous research highlighting belonging as fundamental to positive identity development (11).

Finally, identity was shown to be an evolving process shaped by life transitions and turning points, such as diagnosis or changes in educational settings. While diagnosis often generated uncertainty or anxiety for families, it was gradually incorporated into personal narratives—not as a fixed label, but as a relational framework for understanding oneself in interaction with the environment. As noted by Todd & Shearn (48), knowledge of the diagnosis constitutes a biographical disruption, at least from the family’s perspective.

Regarding the use of LSW, it proves particularly valuable in making visible these complex and multifaceted identities. By foregrounding participants’ voices, LSW facilitated the emergence of counter-narratives that challenge deficit-based or tragedy-centered representations of disability (9, 33). Rather than reproducing externally imposed identities, participants actively positioned themselves as agents with preferences, emotions, and life trajectories, underscoring the ethical and epistemological relevance of narrative approaches when working with people with ID (30). These findings underscore the importance of prioritizing the internal perspectives of individuals with ID in both research and practice, recognizing their capacity to narrate, interpret, and give meaning to their own lives (2). Avoiding reductive identity constructions is therefore not only an ethical imperative, but also a necessary condition for producing more inclusive and socially just knowledge.

The use of LSW enriched the narrative methodology by incorporating visual artefacts, personal objects, and collaborative timeline construction, which supported memory recall and participation beyond oral fluency. Unlike interview-based narrative methods alone, LSW facilitated multimodal expression and co-authored narrative products, enhancing inclusivity while preserving narrative coherence.

Beyond confirming existing theoretical perspectives on narrative identity, this study contributes to the field by showing how life stories can become spaces of resistance to dominant disability discourses. By placing everyday experiences, relationships, and personal meanings at the center, participants’ narratives challenge deficit-oriented views of disability and make visible alternative ways of constructing identity grounded in agency, a sense of belonging, and relational processes. In this way, life stories do not merely reflect identity but actively contribute to its construction, highlighting their value as both a methodological and transformative tool in research guided by inclusive principles.

### 4.1 Limitations

This study presented several methodological and ethical challenges. The diversity of participants required tailoring data collection and analysis to each individual, allowing for a wide diversity of experiences but complicating the systematization of the process. As researchers, we sought to maintain an active, respectful, and person-centered listening, while recognizing that the mediation of support professionals and the research team inevitably introduced interpretive layers that may have shaped how narratives were understood. The combination of personal and family narratives, sometimes divergent, raised analytical and ethical dilemmas related to the plurality of voices and potential conflicts of meaning. Although the study centers on the narrative agency of people with intellectual disability, family interviews introduced complementary voices that provided contextual information not recalled by participants. While this enriches the completeness of the life stories, it also means that some sections may be influenced by perspectives other than those of the protagonists. This reliance on secondary informants may partially dilute the centrality of participants’ own voices.”

Moreover, the narrative nature of the data—often fragmented, changing, or contradictory—reflected the complexity of memory and identity construction. Efforts were made to avoid reducing participants’ identities to the label of “person with a disability,” acknowledging instead their multiple roles and dimensions. Finally, this study underscores the need for clear ethical protocols on informed consent, narrative ownership, and the use of life stories in research with people with ID, an area where although participatory methodological guidance is expanding, further clarification is needed regarding ethical management of co-constructed life stories.

An additional limitation is that the requirement for oral communication excluded individuals with severe communication difficulties.

## 5 Conclusions

This study set out to explore identity construction among individuals with intellectual disabilities through Life Story Work (LSW). The findings show that participants actively engaged in the narrative process and were able to articulate personally meaningful experiences using multimodal resources such as photographs, objects, and collaborative timelines. LSW provided an accessible structure through which participants could organize and express their autobiographical accounts.

The life stories revealed identity-related narratives centered on belonging, roles, aspirations, emotional experiences, and responses to social expectations. These narratives illustrate how participants make sense of themselves and negotiate their identities relationally. The cross-case analysis further identified overarching themes and narrative structures that cut across individual stories, offering insight into shared dimensions of identity construction.

Taken together, the findings suggest that identity construction among individuals with intellectual disabilities is relational, evolving, and embedded in everyday interactions. LSW not only facilitates the articulation of these identities but also creates dialogical spaces in which participants can reclaim meaning, resist limiting discourses, and position themselves as active agents in their own narratives.

Beyond its empirical findings, this study contributes methodologically by demonstrating the value of integrating thematic and narrative analysis within Life Story Work. Conceptually, it reinforces the understanding of identity as a relational and temporally evolving construction shaped through processes of social recognition. Practically, the findings highlight the importance of creating narrative spaces that enable individuals with intellectual disabilities to articulate, negotiate, and re-signify their life experiences. While the study does not aim for statistical generalization, it offers analytically transferable insights that may inform inclusive narrative practices and support more participatory approaches to identity work in socio-educational contexts.

## Data Availability

The raw data supporting the conclusions of this article will be made available by the authors, without undue reservation.

## Footnotes

1 A self-contained classroom refers to a specialized classroom within a mainstream school where students with disabilities receive targeted educational support.
