# Supplementary material for: Life Story Work as an inclusive methodology to explore identity in intellectual disability
Source: Front Psychiatry. 2026 Mar 26;17:1784316. doi: 10.3389/fpsyt.2026.1784316 (PMC13062216; doi:10.3389/fpsyt.2026.1784316)
Supplement: Supplementary file 1 [file DataSheet1.pdf]

Supplementary Table 1. Narrative profiles of participants

Emma	Woman, 23 years old**.** Lives with her parents and older brother. Attended four mainstream schools and one special education school, where she completed a special vocational training program. She is currently enrolled in a day center for adults with disabilities, where she will be able to remain until retirement. Diagnosis: ID.
Mael	Man, 25 years old. Lives with his parents and has a very close relationship with his sister, who lives nearby. Attended preschool and primary education in a mainstream school, then transferred to another mainstream school for two years. He completed secondary education in a special education school and later pursued a special vocational training program. He is currently enrolled in an occupational center. Diagnosis: Down syndrome
Sofia	Woman, 16 years old. She is the youngest of three siblings. Lives with her parents and one of her brothers; the other is attending university in another city. She completed primary education in a mainstream school (in a self-contained classroom), and upon transitioning to secondary education, she moved to her current school. She is in a self-contained classroom, with other students with neurodevelopmental disorders. Diagnosis: ID
Elias	Boy, 14 years old. Lives with his parents, his twin sister, and his younger brother. He is currently enrolled in a mainstream school, attending Middle School, in a self-contained classroom. Diagnosis: pervasive developmental disorder (PDD), as reported by the family. His profile would likely fall under Autism Spectrum Disorder (ASD), and ID although he has not been formally diagnosed as such.
Maya	Girl, 14 years old. Lives with her parents and her older sister (16 years old). She is currently attending Middle School in a mainstream setting, placed in a self-contained classroom. Diagnosis: 8p23 deletion syndrome, a rare genetic condition associated with developmental delays and learning difficulties.
Max	Boy, 16 years old. Lives with his parents. He is currently attending Middle School in a mainstream school, placed in a self-contained classroom. Diagnosis: Williams syndrome, formally diagnosed at age 13. Prior to that, he had been diagnosed with *unspecified developmental disorder* and *mild intellectual disability*. Throughout his educational journey, he has attended four different schools, including one special education center.
Leo	Boy, 13 years old. Lives with his parents and his sister, along with a cat and a dog. He is currently attending Middle School in a mainstream school, placed in a self-contained classroom. Diagnosis: ASD and ID. He has expressed a strong interest in studying history at university. His current school is considering transitioning him to a general education classroom with peers without disabilities.

Eva	Woman, 32 years old. Lives in a supported living apartment and attends a day center for people with intellectual disabilities. Her mother visits her on weekends and they spend holidays together. In the past, she worked in a sheltered employment center. Over the years, she has received various diagnoses, including autism, ID, and oppositional defiant disorder
